# Effectiveness and adherence to closed face shields in the prevention of COVID-19 transmission: a non-inferiority randomized controlled trial in a middle-income setting (COVPROSHIELD)

**DOI:** 10.1186/s13063-022-06606-0

**Published:** 2022-08-20

**Authors:** Andrea Ramirez Varela, Alejandro Pacheco Gurruchaga, Silvia Restrepo Restrepo, Juan David Martin, Yessica Daniela Campaz Landazabal, Guillermo Tamayo-Cabeza, Sandra Contreras-Arrieta, Yuldor Caballero-Díaz, Luis Jorge Hernandez Florez, John Mario González, Juan Carlos Santos-Barbosa, José David Pinzón, Juan José Yepes-Nuñez, Rachid Laajaj, Giancarlo Buitrago Gutierrez, Martha Vives Florez, Janner Fuentes Castillo, Gianni Quinche Vargas, Andres Casas, Antonio Medina, Eduardo Behrentz, on behalf of the CoVIDA Working Group, Yenny Paola Rueda Guevara, Daniela Rodriguez Sanchez, Marcela Guevara-Suarez, Marylin Hidalgo, Paola Betancourt

**Affiliations:** 1 Faculty of Medicine, Universidad de los Andes, Cra 7 #116-05, Bogotá, 11001000 Colombia; 2 United Nations Development Programme (UNDP), Bogota, Colombia; 3 Department of Food and Chemical Engineering, Universidad de los Andes, Bogotá, Colombia; 4 Laboratory of Biomedical Sciences (CBMU), Universidad de los Andes, Bogotá, Colombia; 5 Independent consultant in geographic data, COVPROSHIELD, Bogota, Colombia; 6 Faculty of Economics, Universidad de los Andes, Bogotá, Colombia; 7 Clinical Research Institute, Universidad Nacional de Colombia, Bogotá, Colombia; 8 Department of Biological Sciences, Universidad de los Andes, Bogotá, Colombia; 9 Center for Social Norms and Behavioral Dynamics at The University of Pennsylvania, Philadelphia, USA; 10 Yale Center for Engineering Innovation & Design, New Haven, USA; 11 Universidad de los Andes, Bogotá, Colombia

**Keywords:** Clinical trial, SARS-CoV-2, COVID-19, Closed face shield, Face mask

## Abstract

**Background:**

The use of respiratory devices can mitigate the spread of diseases such as COVID-19 in community settings. We aimed to determine the effectiveness of closed face shields with surgical face masks to prevent SARS-CoV-2 transmission in working adults during the COVID-19 pandemic in Bogotá, Colombia.

**Methods:**

An open-label non-inferiority randomized controlled trial that randomly assigned participants to one of two groups: the intervention group was instructed to wear closed face shields with surgical face masks, and the active control group was instructed to wear only surgical face masks. The primary outcome was a positive reverse transcription polymerase chain reaction test, IgG/IgM antibody test for SARS-CoV-2 detection, or both during and at the end of the follow-up period of 21 days. The non-inferiority limit was established at − 5%.

**Results:**

A total of 316 participants were randomized, 160 participants were assigned to the intervention group and 156 to the active control group. In total, 141 (88.1%) participants in the intervention group and 142 (91.0%) in the active control group completed the follow-up. Primary outcome: a positive SARS-CoV-2 test result was identified in one (0.71%) participant in the intervention group and three (2.1%) in the active control group. In the intention-to-treat analysis, the absolute risk difference was − 1.40% (95% CI [− 4.14%, 1.33%]), and in the per-protocol analysis, the risk difference was − 1.40% (95% CI [− 4.20, 1.40]), indicating non-inferiority of the closed face shield plus face mask (did not cross the non-inferiority limit).

**Conclusions:**

The use of closed face shields and surgical face masks was non-inferior to the surgical face mask alone in the prevention of SARS-CoV-2 infection in highly exposed groups. Settings with highly active viral transmission and conditions such as poor ventilation, crowding, and high mobility due to occupation may benefit from the combined use of masks and closed face shields to mitigate SARS-CoV-2 transmission.

**Trial registration:**

ClinicalTrials.gov NCT04647305. Registered on November 30, 2020

**Supplementary Information:**

The online version contains supplementary material available at 10.1186/s13063-022-06606-0.

The disease caused by the severe acute respiratory syndrome coronavirus 2 (SARS-CoV-2) or COVID-19 has caused more than 3.1 million deaths globally as of May 2, 2021, and more than 152 million cases have been reported worldwide [1]. Latin America has been one of the epicenters of the pandemic since July 2020 [2–4]. Estimates have shown that there will be more than 1.1 million deaths in the region by the second half of 2021. Colombia is one of the five most affected Latin American countries, exceeding 2.8 million cases and more than 73,000 deaths [1, 5].

Latin America has experienced rapid economic growth during the last decade, and it is the most urbanized region in the developing world, with approximately 80% of the population living in urban areas. As this number increases and population density increases in countries like Colombia, the number of people demanding jobs and opportunities also grows, with increasing rates of informal employment [6, 7]. These conditions make Latin America and Colombia settings in which the SARS-CoV-2 pandemic could be difficult to control and mitigate, as demonstrated by the low rates of lockdown adherence, slow rates of vaccination, overcrowding in public transportation, and increased mobility in urbanized areas. Therefore, the risk of exposure to the virus and infection in adults circulating in the city is likely high.

SARS-CoV-2 is primarily transmitted airborne via respiratory droplets or aerosols generated when an infected person coughs or sneezes; to a lesser extent, contact transmission is possible through fomites [8–12]. Most public health agencies worldwide recommend using face masks (i.e., surgical masks, cloth masks, or respirators), physical distancing, and handwashing among other non-pharmacological interventions to prevent SARS-CoV-2 transmission [13–16]. Surgical face masks have proven to be one of the most effective measures (97.5% efficacy vs. over 99.9% for fitted N95 masks) against the transmission of SARS-CoV-2 infection in hospital and community settings [16–21].

There is promising evidence showing that the use of eye and face protection, such as closed face shields, confers an extra benefit against airborne diseases like COVID-19 by preventing contact between face and hands, protecting the mucous membranes of the face (e.g., eyes), and blocking the airflow with infected particles from reaching the face [16, 22–26]. A systematic review and meta-analysis suggested that eye protection (described as the use of face shields and/or goggles) was associated with a decrease of 78% in the incidence of COVID-19 (*OR* = 0.22, 95% CI [0.12, 0.39]) in health care and non-health care (e.g., community) settings [16].

Since August 2020, Colombia has allowed economic reactivation activities, and people’s mobility has been increasing [27]. In the same period, a rapid increase in COVID-19 cases has been observed, including the worst pandemic peaks.

Additional individual measures to prevent SARS-CoV-2 infection have been proposed to mitigate the pandemic. In 2020, the United Nations Development Programme (UNDP) and the Colombia Makers community developed a closed face shield prototype to prevent SARS-CoV-2 infection called “Cascos de Vida” (https://www.undp.org/es/colombia/cascos-de-vida). In collaboration with Yale University, this development was assessed with a visualized computational analysis of the aspects of the closed face shield design that most effectively mitigates the blockage of air currents around the face. As a prototype, this face shield performed positive air current blockage, mainly due to the broad width of its front. Also, this device generates a behavior change to prevent people from touching their faces with their hands and to raise awareness about self-care and protection [28].

The use of face shields in a city such as Bogotá could provide significant protection for populations with a high risk of contagion due to their occupations [29]. This includes those who work in closed environments with poor ventilation or crowded environments where it is difficult to keep a safe distance.

However, all medical devices, regardless of class and risk, must be evaluated for clinical performance and benefit in real-life settings [30]. Clinical trials are suggested to evaluate the effectiveness of personal protective equipment (PPE) such as face shields [31]. To our knowledge, no randomized controlled trials have been carried out in Colombia or in Latin America to assess the effectiveness of closed face shields as protective measures against COVID-19 in working adults. This study aimed to determine the effectiveness of and adherence to using closed face shields with surgical face masks compared to using only surgical face masks to prevent SARS-CoV-2 transmission in adults in Bogotá, Colombia.

## Methods

### Study design

COVPROSHIELD (ClinicalTrials.gov ID: NCT04647305) was an open-label non-inferiority randomized controlled trial nested within the CoVIDA project. The CoVIDA project was an observational epidemiological study framed within an intensified sentinel epidemiological surveillance strategy. Using SARS-CoV-2 screening, the CoVIDA project enrolled over 58,000 participants that, due to their occupations, had higher mobility and were at an increased risk of infection in Bogotá, Colombia, such as health and essential services workers [29, 32]. This study is reported according to the Consolidated Standards of Reporting Trials (CONSORT) statement (S1 CONSORT checklist).

### Participants

The study included participants from the CoVIDA project that were enrolled during November and December 2020, 18 years or older, and with a negative reverse transcription polymerase chain reaction (RT-PCR) test for SARS-CoV-2 in the previous 2 months. Within this group, eligible participants included those who (a) lived in a geographic area with active COVID-19 transmission (defined by the number of cases reported locally) and in areas with medium, medium-high, and high vulnerability index (higher prevalence of comorbidities and social and economic vulnerabilities, determined by the Colombian National Statistics Department, DANE) and (b) worked outside their homes for at least 2 days during the last week [33]. The non-inclusion criteria were retirement, unemployment, home-based working, history of laboratory-confirmed COVID-19, working in health care, and daily N95 mask or face shield use. Recruitment and enrollment in the trial were conducted over the phone. Participants provided informed consent verbally, and complete information about the trial was sent via email.

### Randomization and allocation

Considering an allocation ratio of 1:1, random blocks of sizes 2, 4, and 6 were used to assign participants to one of the two study arms. Block randomization was implemented to ensure that comparison groups would be generated according to the predetermined ratio. This study was an open trial; hence, no blinding of the intervention was applied. Different data collection team members carried out the sequence of randomization, assignment to the intervention, and implementation.

Participants who accepted to join the trial underwent a baseline RT-PCR and serological anti-SARS-CoV-2 test to rule out previous SARS-CoV-2 infection before trial enrollment. Those with negative results were randomized into one of two groups: (a) the intervention group (IG), who were instructed to wear closed face shields with surgical face masks, and (b) the active control group (ACG), who were instructed to wear only surgical face masks (S2 File). Randomization was performed by an independent statistician so that the clinical staff was blinded to the results of the process. A randomization sequence was created using the STATA (version 16.0, StataCorp, College Station, TX, US) with a 1:1 allocation using random block sizes of 2, 4, and 6. Instructions for individual allocation of participants were provided to the clinical data manager by the randomization staff to guarantee allocation concealment. This study was an open trial; hence, no blinding of the intervention was applied.

### Intervention

The follow-up period of 21 days was selected according to the CoVIDA project methods (based on the national guidelines for SARS-CoV-2 screening in high-risk groups) and literature on clinical trials assessing the efficacy of PPE [34]. Once the participant was assigned to one of the two groups, PPE was sent to their home address for each day of participation in the trial (21 surgical face masks for all participants, plus closed face shields for participants in the IG). Participants were instructed to wear the assigned PPE every time they went to work and on daily activities outside the home. All the participants received a recorded educational intervention via email or phone that provided recommendations about COVID-19 prevention measures, guidance to ensure adherence, and appropriate handling of the assigned PPE (S3 File). Verification was performed via email or phone to ensure comprehension of the information provided. The first day of follow-up corresponded to the day the participant would be starting to use the PPE assigned. Follow-up with participants was conducted twice a week by phone. A weekly short questionnaire was performed on days 7, 14, and 21 to evaluate health status, SARS-CoV-2 symptoms, PPE use, and adherence. A pilot was carried out to assess the comprehensiveness of the instrument by the participants (S4 File). During follow-up, participants were requested to provide photographs using the PPE to assess the correctness of use. Participants were excluded if, during the follow-up, they reported an occupation change that may have led to reduced exposure to SARS-CoV-2 infection (e.g., became unemployed, changed from work outside the home to home-based work, or reported daily use of N95 face masks or another face shield). This guaranteed a similar community exposure to the virus in the two arms of the study.

### Laboratory sample collection

For the detection of the SARS-CoV-2 virus, an RT-PCR test by nasopharyngeal swab was performed using the U-TOP™ COVID-19 detection system [35]. For the qualitative antibody test for SARS-CoV-2, serum and the Roche Laboratories SARS-CoV-2 Rapid Antibody Test® were used, which detects the presence of anti-SARS-CoV-2 IgM/IgG. The test’s accuracy parameters are described elsewhere [36].

### Outcomes

The primary outcome was the composite result of positive RT-PCR or seroconversion during follow-up. If the participant reported symptoms and/or epidemiological nexus compatible with the case definition for COVID-19 according to the public health surveillance of Colombia [37] (S5 File) during follow-up, they underwent an RT-PCR test to rule out the infection. If the RT-PCR test was positive, the follow-up ended and the contact tracing strategy was activated. Finally, both RT-PCR and blood antibody tests were taken at the end of the follow-up to rule out SARS-CoV-2 infection. Therefore, the effectiveness or benefit of the intervention is to prevent SARS-CoV-2 infection, providing additional protection to high-risk workers.

Secondary outcomes including PPE use variables such as number of hours per day and days per week using the assigned PPE and reasons for no use were also collected. Device use was assessed weekly and globally at the end of the study. Additional categories were established to analyze the adherence variable after the start of the trial to explain the secondary outcome in more detail. Weekly adherence was classified into three categories: complete adherence, partial adherence, and no adherence to the interventions. The categories are explained in S6 File.

Complete adherence was defined as the proper daily use of the assigned PPE (every time the participant left their home, removed only for eating or while alone). The closed face shield had to be washed or disinfected with alcohol daily, and the surgical face mask had to be changed every day.

The definition of partial adherence included using the assigned PPE whenever participants went out but removing it for a reason other than those recommended. Partial adherence also included proper use of the assigned PPE but without cleaning/changing the devices appropriately.

Non-adherence was defined as using the assigned PPE whenever participants went out but removing it for a reason other than those recommended and not properly cleaning/changing the devices. Non-adherence also included participants leaving their homes without using the assigned PPE.

Based upon the three weekly follow-up scores, a global adherence variable was defined, and five categories were obtained: high adherence, medium-high adherence, medium adherence, medium-low adherence, and low adherence (S6 File).

PPE use was evaluated according to the number of days going out of the home to work and other activities, number of times the participant removed the PPE, and the average daily hours using the devices.

### Statistical analysis

Sample size calculation was performed considering a significance level (alpha) of 5%, power (1-beta) of 80%, and a non-inferiority limit of − 5%. For an actual difference in favor of the experimental treatment, a margin of 2% (97% of success for the IG vs. 95% of success for the ACG) was established. The study required 144 participants (97 per arm). Anticipating 20% of the loss to follow-up, 232 were considered for recruitment (116 per arm).

Cumulative COVID-19 incidence was calculated as the number of new cases at follow-up. This was defined by the number of participants with laboratory evidence of infection, either with a positive result for RT-PCR test, IgG/IgM antibody test for SARS-CoV-2 detection, or both during and at the end of follow-up.

Baseline characteristics were reported using absolute and relative frequencies. Median and interquartile ranges (IQR) were reported for quantitative variables. The effectiveness of the face shield with a surgical face mask was assessed by non-inferiority analysis relative to the surgical face mask alone. We analyzed the outcome of effectiveness, estimating the difference in the cumulative incidence of SARS-CoV-2 infection (RT-PCR or IgG/IgM antibody test) and the two-sided 95% confidence interval (CI). We established a non-inferiority limit of − 5% considering the literature on the effectiveness of other respiratory devices (90% cloth mask vs. 95% surgical mask) [38]. Non-inferiority for the closed face shield was achieved if the absolute risk reduction was greater than the prespecified non-inferiority limit. The results were estimated with both an intention-to-treat and per-protocol analysis. The intention-to-treat analysis included all patients allocated and continued to fulfill the inclusion criteria during the follow-up. Data for participants with no results for the RT-PCR or IgG/IgM antibody test for SARS-CoV-2 were imputed with negative results, given the low incidence of the primary outcome in both groups. The per-protocol analysis was carried including all participants with complete follow-up, RT-PCR test, and IgG/IgM antibody test for SARS-CoV-2. In the case of non-inferiority, a superiority limit of + 2% (considering an efficacy of 97% of the closed face shield and 95% of the surgical face mask) was established.

Finally, a post hoc analysis was conducted using the adherence information under three scenarios (including only data of participants with complete follow-up). The first one included only the participants who reported complete adherence to the assigned PPE. The second included those with high or medium adherence, and the third included those with high, medium-high, and medium adherence (S8 Table). Differences in input characteristics were evaluated using Fisher’s exact tests for categorical variables and Mann–Whitney *U* test for continuous variables, as appropriate. A *p*-value < 0.05 was considered statistically significant. Statistical analyses were performed in STATA (version 16.0, StataCorp, College Station, TX, US).

### Ethical aspects

The study was evaluated by the ethics committee of Universidad de los Andes for approval (Act No. 1278 of 2020). Trial registration: ClinicalTrials.gov ID: NCT0464730

## Results

### Participants

**Fig. 1:**
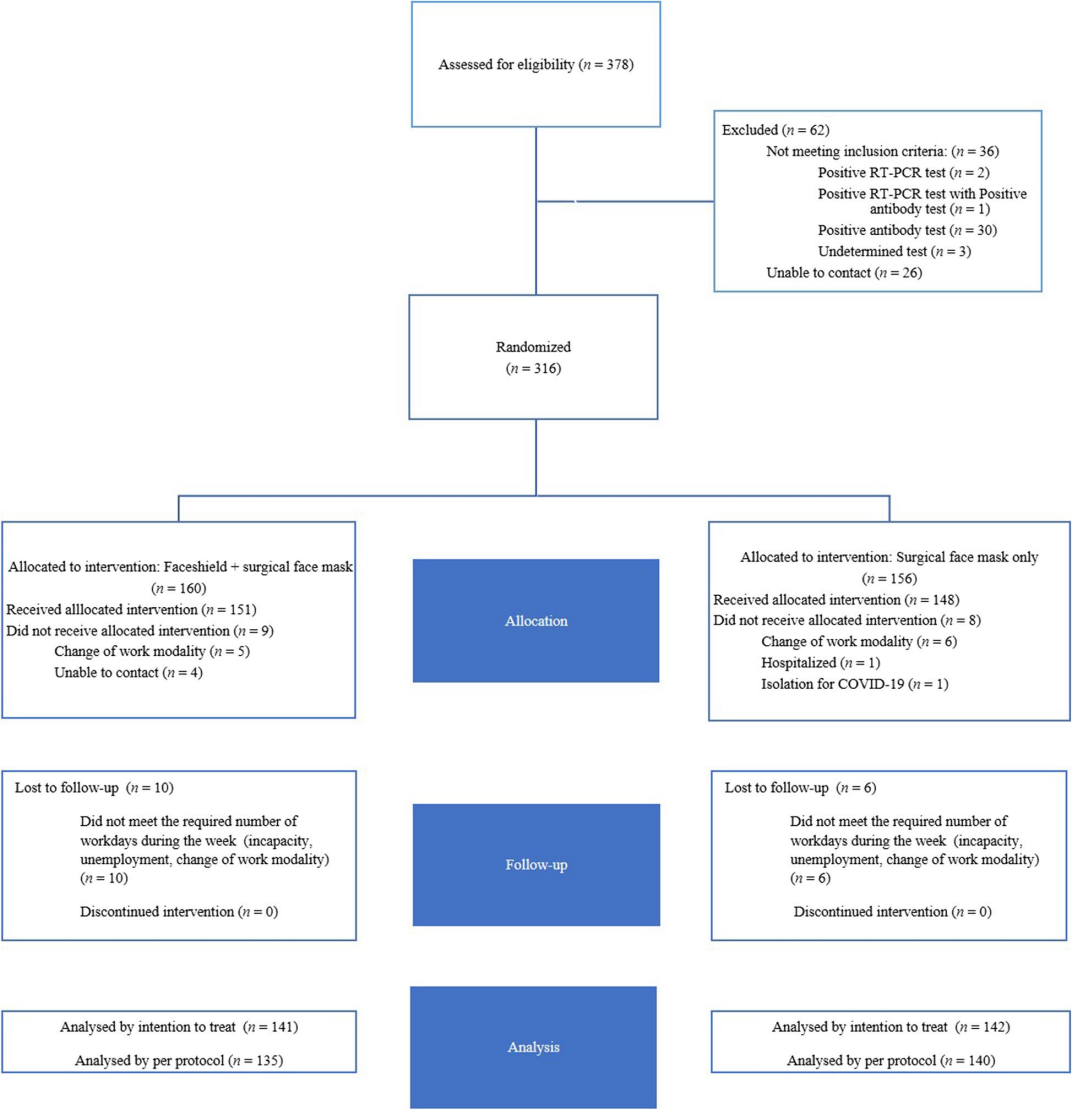
Study flowchart

The study was conducted from January 12 to March 13, 2021. Enrollment of participants was carried out between January 12 and January 22, and 2001 participants from the CoVIDA project were invited to participate in the study. A total of 378 participants met the inclusion criteria and agreed to take the baseline RT-PCR and antibody tests. A total of 352 participants underwent the laboratory tests. Twenty-four of them had positive IgG antibody tests, and six had positive IgG and IgM tests. One participant had both positive RT-PCR test and IgG antibody test, for a baseline seroprevalence of 8.8%. Only two participants had a positive RT-PCR test alone, and three participants had an undetermined result and did not repeat the test (Fig. 1).

**Fig. 2:**
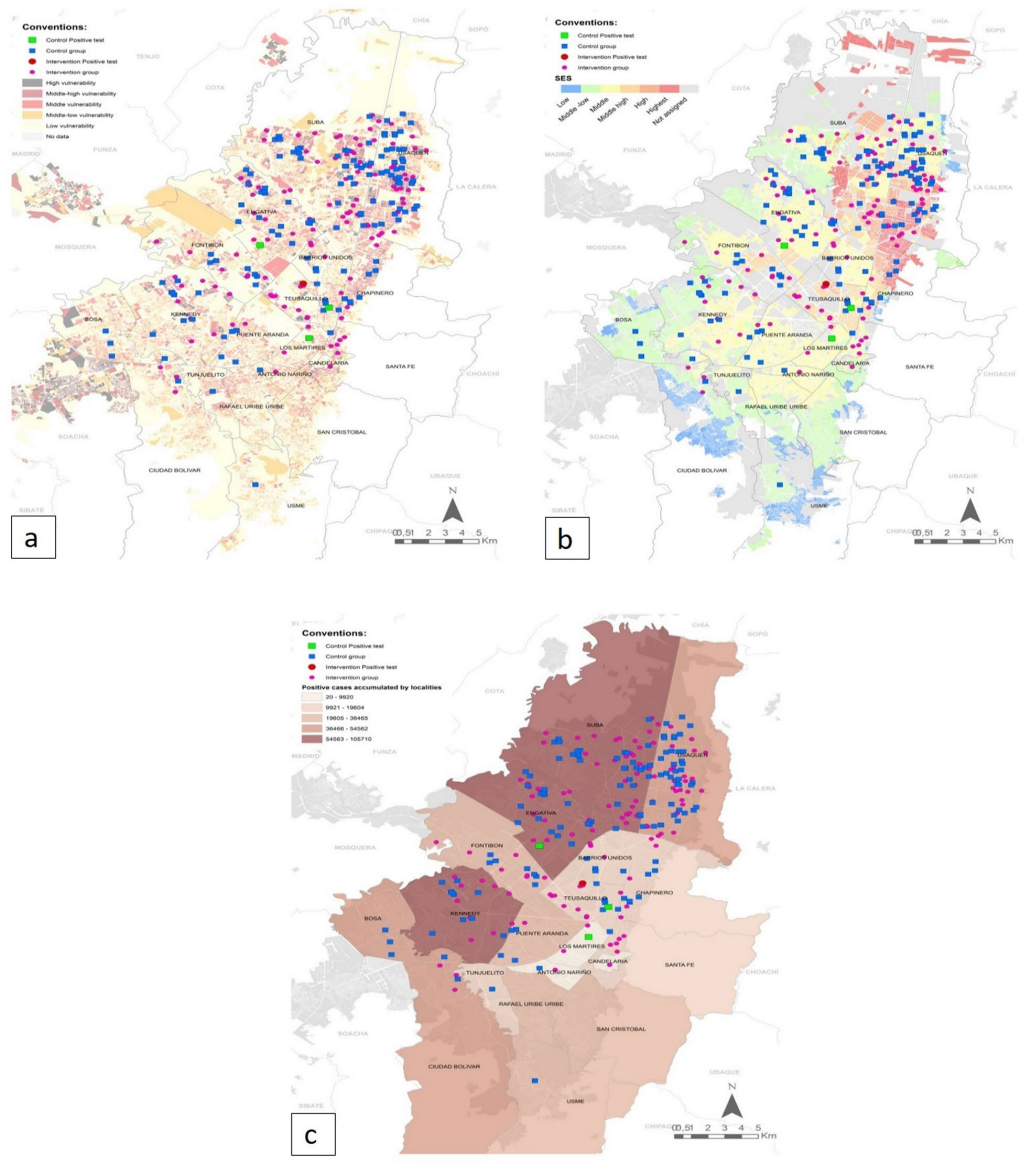
Distribution of study participants according to socioeconomic stratum, vulnerability index, and residence localities. **a** Vulnerability index. **b** Socioeconomic stratum. **c** Residence localities

A total of 316 participants were randomized. A total of 160 participants were allocated to the IG (face shield and surgical face mask), and 156 were allocated to the ACG (surgical face masks only). A total of 135 (84.4%) participants of the IG and 140 (89.7%) of the ACG completed the follow-up (Table 1, Fig. 2). Baseline characteristics regarding socioeconomic strata, vulnerability index, and residence location were similar between the groups (Fig. 2). The median age was 36 years (min 30, max 48), and 140 (49.5%) were female. The most common occupation among all participants was office worker, with 164 (57.9%) participants. The number cohabiting with three or fewer people was 90 participants (63.8%) in the IG and 99 participants (66.7%) in the ACG. A total of 108 (76.6%) participants in the IG were classified as middle and middle-high on the vulnerability index, while 106 (74.6%) participants in the ACG were classified into these categories (Table 1).

**Table 1:** Baseline characteristics of the study population

	Total (*N* = 283)	Face shield + surgical face mask group (*N* = 141)	Surgical face mask group (*N* = 142)
***Sociodemographic characteristics***			
Sex			
Female, *n* (%)	140 (49.5)	68 (48.2)	72 (51.4)
Age			
Median (IQR)	36 (30–48)	36 (29–50)	36 (30–47)
Socioeconomic stratum^a^			
Very low	1	1 (0.7)	0 (0.0)
Low	19	8 (5.6)	11 (7.7)
Middle-low	108	53 (37.6)	55 (38.7)
Middle	108	52 (36.9)	56 (39.5)
Middle-high	29	17 (12.1)	12 (8.5)
High	18	10 (7.1)	8 (5.6)
Type of health insurance			
Contributive, special, exception	267	131 (92.9)	136 (95.7)
No affiliated, no determined	11	7 (4.9)	4 (2.8)
Subsidiary	5	3 (2.1)	2 (1.4)
Number cohabiting			
≤ 3	189	90 (63.8)	99 (66.7)
> 3	94	51 (36.2)	43 (30.3)
Vulnerability index			
Low	4	3 (2.1)	1 (0.7)
Middle-low	5	4 (2.8)	1 (0.7)
Middle	110	51 (36.2)	59 (41.5)
Middle-high	104	57 (40.4)	47 (33.1)
High	60	26 (18.4)	34 (23.9)
***Occupation, n*(%)**			
Office employees	164 (57.9)	79 (56.0)	85 (59.9)
Public transportation drivers	28 (9.9)	12 (8.5)	16 (11.3)
Salesperson/cashiers/shop employees	18 (6.4)	12 (8.5)	6 (4.2)
Non-hospital health care workers	16 (5.6)	9 (6.4)	7 (4.9)
Workers with a high load of physical activity (builders, mechanics, physical trainers)	12 (4.2)	3 (2.1)	9 (6.3)
Teachers (including school and university)	10 (3.5)	8 (5.7)	2 (1.4)
Journalists	8 (2.8)	5 (3.5)	3 (2.1)
Deliverymen/couriers	5 (1.7)	4 (2.3)	1 (0.7)
Others (hairdressers, farmers, armed forces, cooks, caregivers, domestic employees)	22 (7.7)	9 (6.4)	13 (9.1)

^a^Socioeconomic strata as defined by the National Department of Statistics (DANE) of Colombia: 1 (*very low*) to 6 (*high*)

A total of 7 (4.9%) participants were identified as suspected COVID-19 cases in the ACG. These participants were primarily women (*n* = 5, 71.4%) and office workers (*n* = 5, 71.4%). On the other hand, 6 (4.2%) participants in the IG were identified as suspected COVID-19 cases. These participants were primarily women (*n* = 4, 66.7%) and office workers (*n* = 5, 83.3%). However, none of the suspected cases tested positive. The symptoms reported by these participants are listed in S7 Table.

### Primary outcome

At the end of the study, the primary outcome was identified in 1 (0.71%) participant in the IG, who was a public transportation driver, and 3 (2.1%) of the participants in the ACG, two women and one man, who were office workers. Seroconversion was only observed in 2 (1.4%) cases in the ACG.

**Fig. 3:**
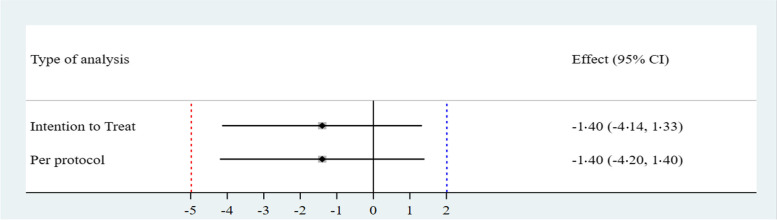
Comparison of the results of the intention-to-treat and per-protocol analyses

In the intention-to-treat analysis, the absolute risk difference was − 1.40% (95% CI [− 4.14%, 1.33%]); in the per-protocol analysis, the absolute risk difference was − 1.40% (95% CI [− 4.20%, 1.40%]), indicating non-inferiority of the closed face shield with surgical face mask in both analyses (Table 2, Fig. 3). RT-PCR and seroconversion results are found in Table 2. Non-significant differences were found in the distribution of positive test results for any comparisons (Table 2).

**Table 2:** Comparison of the primary outcome between the groups

	Face shield + surgical face mask group, ***N*** (%)	Surgical face mask group, ***N*** (%)	Absolute risk difference	***p***-value^**a**^
**Intention-to-treat analysis**	**(*N*= 141)**	**(*N*= 142)**	**% [95% CI]**	
Primary composite outcome	1 (0.7)	3 (2.1)	− 1.40 [− 4.14, 1.33]	0.31
Positive RT-PCR test	1 (0.7)	1 (0.7)	0.005 [− 1.94, 1.96]	0.74
Positive antibody test				
IgG	0 (0)	2 (1.4)	− 1.40 [− 3.3, 0.53]	0.25
IgM	0 (0)	0 (0)	**–**	**–**
**Per protocol analysis**	**(*N*= 135)**	**(*N*= 140)**	**% [95% CI]**	
Primary composite outcome	1 (0.7)	3 (2.7)	− 1.40 [− 4.20, 1.40]	0.32
Positive RT-PCR test	1 (0.7)	1 (0.7)	0.02 [− 1.98, 2.02]	0.74
Positive antibody test				
IgG	0 (0)	2 (1.4)	− 1.40 [− 0.39, 5.37]	0.25
IgM	0 (0)	0 (0)	**–**	**–**

*RT-PCR* reverse-transcriptase polymerase chain reaction

^a^*p*-value calculated with the Fisher's exact test

**Fig. 4:**
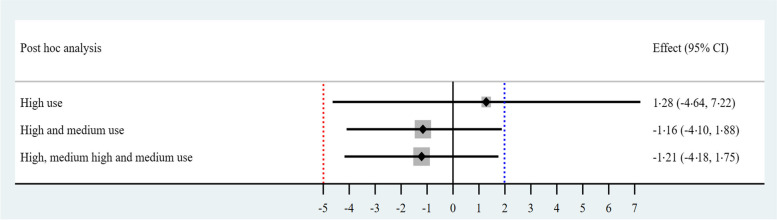
Post hoc analysis

When discriminating by the level of adherence, the non-inferiority margin was achieved in all analyses, including high adherence participants alone (1.28%, 95% CI [− 4.64%, 7.22%]), high and medium adherence (− 1.16%, 95% CI [− 4.10%, 1.88%]), and medium-high and medium adherence (− 1.21%, 95% CI [− 4.18, 1.75]; Fig. 4, S8 Table).

### Secondary outcomes

The number of working days was similar in both groups (12 vs. 13, *p* = 0.46), and the median number of days of PPE use was higher in the ACG (*p* < 0.001). The hours of use of the assigned PPE were higher in the ACG. The number of hours using face masks was higher in the ACG (Table 3).

**Table 3:** Comparison of the use of the intervention between groups

	**Total (*N*= 275), median, IQR**	**Face shield + surgical face mask group (*N*= 135), median, IQR**	**Surgical face mask group (*N*= 140), median, IQR**	***p*-value**
***Mobility***				
Number of working days during the follow-up period	12 (9–15)	12 (8–15)	13 (9–15)	0.46ª
***PPE adherence***				
Average number of days of use of assigned PPE	15 (12–17)	14 (11–17)	15 (13–18)	< 0.001ª
Average number of hours of use of the assigned PPE per day	6 (4.3–8)	5 (4–6.7)	7.3 (5.3–8.7)	< 0.001ª
Average number of hours of use of face mask only per day	6 (4–8)	5.5 (4–8)	7.3 (5.3–8.7)	< 0.001ª
	***n*(%)**	***n*(%)**	***n*(%)**	
***Adherence use of the intervention***				< 0.001^b^
High	161 (58.5)	37 (27.4)	124 (88.6)	
Medium-high	78 (28.4)	63 (46.7)	15 (10.7)	
Medium	7 (2.5)	7 (5.2)	0 (0)	
Medium-low	21 (7.6)	20 (14.8)	1 (0.7)	
Low	8 (2.9)	8 (5.9)	0 (0)	

Medians and % were calculated based on the participants who provided complete information during the three follow-up calls. Six participants (4.2%) are missing values among the intervention group, and two (1.4%) are missing values among the active control group

*IQR* interquartile range, *PPE* personal protective equipment

^a^*p*-value calculated with the Mann–Whitney *U* non-parametric test

^b^*p*-value calculated with the Fisher's exact test

Higher adherence was reported in the ACG compared to the IG. The participants who reported higher adherence were, on average, older than the ones reporting lower adherence, aged 36 years (IQR 30–48) and 31 years (IQR 27.5–41), respectively. In the high adherence group, 52.2% (*n* = 84) of the participants belonged to a middle or above middle (middle-high and high) socioeconomic stratum; 51.5% (*n* = 83) of the participants were men. In the lower adherence group, 62.5% (*n* = 5) of the participants belonged to the middle-low or lower (low and very low) socioeconomic stratum, and 50% (*n* = 4) of the participants were men. No statistically significant differences were found for any of these variables.

In the ACG, almost all the participants reported high or medium-high adherence to the intervention: 124 (88.6%) and 15 (10.7%), respectively. In the IG, only 37 (27.4%) reported high adherence, and 63 (46.7%) reported medium-high adherence (Table 3). Forty-one percent of the ACG participants reported removing the surgical face mask while being outside the home. The main self-reported reason was for eating and drinking. This proportion was 37.1% for the face shield and 38.8% for the face mask in the IG. The main self-reported reasons for removing the face shield were eating and drinking, to rest from the discomfort produced by wearing the PPE (heat, lack of visibility, fogging), and being alone. Reasons for removing the face mask in this group included eating or drinking and to rest (data not presented in tables). During weekdays, the main self-reported reason for not wearing the face shield was lack of visibility. For the weekends, the reasons given were to exercise and be at family or friends’ reunions.

No participants in the IG or ACG reported not using the face mask while being outside the home.

## Discussion

To the authors’ knowledge, this is the first randomized controlled trial that aimed to evaluate the effectiveness and adherence to closed face shields with surgical face masks to prevent SARS-CoV-2 transmission in a working population with high mobility and exposure outside health care settings during the COVID-19 pandemic. The study’s main finding was that the laboratory-confirmed SARS-CoV-2 infection was lower in the group that wore closed face shields with surgical face masks compared to the group that wore surgical face masks only. Four participants had a confirmed SARS-CoV-2 infection, three of them in the ACG. Although no statistically significant differences were observed in the incidence of SARS-CoV-2 between the two groups given the low incidence of the event, we believe that there is a clinical, social, and public health significance if at least three COVID-19 cases were prevented with this intervention, especially if these cases would have had a severe or even fatal outcome.

Even though the surgical face mask protects from the inhalation of respiratory droplets, which is the primary way of transmission of SARS-CoV-2 [11, 12], there is enough evidence that suggests that infection may also occur through contact with aerosols, fomites with the respiratory airways, and the mucous membranes of the face, such as the eyes [9, 11, 12, 39, 40]. In this scenario, the use of face masks exclusively may not provide enough protection. Also, eye protection has been associated with lower COVID-19 incidence in health care settings [16]. This finding is supported by the fact that face shields are effective in the context of other respiratory viruses, such as influenza [41]. The additional protection conferred by the closed face shield may have prevented a higher incidence of SARS-CoV-2 in the ACG.

Adherence plays an important role in the effectiveness of interventions related to PPE use. The present study was carried out during the second peak of the pandemic in Bogotá. Hence, the recommendation of wearing face masks every time at any place was highly promoted by the government. This could have contributed to higher adherence in the ACG than in the IG, where participants reported removing the face shield and keeping only the surgical face mask [16, 22, 42, 43]. Regarding the effect of adherence on the effectiveness of PPE, several studies carried out in the community have shown that lower adherence during epidemics of airborne infectious diseases such as influenza [41, 44] or SARS-CoV-2 [45] could directly affect the effectiveness of interventions.

Participants reported a higher feeling of protection when using the face shield in settings where there is relatively little airflow and in crowded places where keeping a safe distance from others is nearly impossible, such as retail stores, private security, construction, restaurants, supermarkets, banks, public transportation, and transportation arranged through rideshare apps. This higher sense of protection in public spaces may be critical in the context of aerosol transmission of SARS-CoV-2 and might be one of the main advantages of using closed face shields [9, 11]. This PPE may benefit high-risk occupations with higher mobility and more direct contact with the public. However, closed face shields must always be used in conjunction with face masks due to the lack of seals in the peripheral areas that can allow penetration of the aerosols [25, 42, 43].

Participants in the IG presented a higher reluctance while using the assigned PPE compared to the ACG. This plays a pivotal role in use, given that this may be relevant when deciding whether or not to use the device. This observation is relevant to young people, who tend to be more sensitive to perceived judgment and, thus, more reluctant to use it [46]. The higher proportions of non-use and removal of the PPE in the closed face shield group may be explained by the higher rates of sense of judgment found in this group, likely exacerbated by the fact that the closed face shield is common in health care settings but not in community settings. Normative behaviors may also play a significant role in adherence beyond the effect of any other individual or group characteristic [47].

Other facts may have limited adherence to the wearing of closed face shields. Among the most common reasons reported for non-adherence to the intervention were sight restrictions, fogging, and heat. These findings are consistent with the discomforts reported by some studies that evaluated the compliance and perceptions of face shields’ implementation for protection against SARS-CoV-2 infection in health care workers [25, 42, 48]. This suggests the importance of studying new materials to achieve more comfort and, therefore, higher use.

Acknowledgment of the additional protection that the closed face shields may confer is necessary to extend their use in the community, ensure easy access, and extend perceptions of usefulness and protection in individuals and families, which finally may determine the willingness of other members of the community to use them [49]. It is desirable that high-quality virus-related information becomes available to all audiences to create positive correlations between knowledge and attitudes towards device use. Literature shows that a greater perception of risk strengthens awareness of self-care, promoting adherence and use of PPE [50–52]. Strategies can be implemented to achieve behavioral changes in the population to obtain compliance with the use of face shields [53].

The strengths of this study include (a) assessment of the primary outcome with both laboratory-confirmed RT-PCR and serological tests, which ensured that the participants included in the study did not present the outcome at the beginning of the study and guaranteed the correct assessment of the main outcome by the end of follow-up; (b) frequent monitoring, which ensured that the participants in both groups were equally exposed to contagion and also enabled low loss to follow-up rates (6.6%, *n* = 10 in the IG; 4%, *n* = 6 in the ACG; Fig. 1); (c) assessment of PPE adherence; and (d) the use of structured questionnaires and scripts prevented interviewer bias. Limitations include the fact that the study was carried out during the second peak of the pandemic in the city; therefore, lockdowns and mobility restrictions were in place and could have changed the participants’ behavior, impacting adherence results, especially in the ACG.

The closed face shields were intended to minimize exposure to infections and allow economic reactivation. They were also conceived for people with high levels of informal labor, poverty, lack of universal social protection, and the inability to work from home, which is frequent in highly urbanized low- and middle-income countries. Countries with highly active viral transmission and lagging rates of vaccination and/or herd immunity can benefit from combining face masks with closed face shields if they succeed in promoting high adherence. Therefore, additional studies using comparable measures and similar settings are advisable to continue generating knowledge about effectiveness with high adherence to closed face shields, given the optimistic preliminary findings in those who showed high adherence in this trial.

## Conclusions

The use of closed face shields with surgical face masks was non-inferior to using the surgical face mask alone to prevent SARS-CoV-2 infection in highly exposed groups in one of the largest cities in Latin America during the second pandemic peak in the country. Although no statistically significant differences were observed in the incidence of SARS-CoV-2 between the two groups given the low incidence, we believe that there is a clinical, social, and public health significance if at least three COVID-19 cases were prevented with this intervention, especially if these cases would have had a severe or even fatal outcome.

Settings with highly active viral transmission and poor ventilation, crowding, and high mobility due to occupation may benefit from the combined use of masks and closed face shields to mitigate SARS-CoV-2 transmission.

## Supplementary Information

**Additional file 1.** CONSORT checklist.

**Additional file 2.** Description of the face shield implemented during the intervention.

**Additional file 3.** Recorded educational intervention during the follow-up period.

**Additional file 4.** Questionnaire to evaluate the state of health and proper use of the intervention during the follow-up period.

**Additional file 5.** Case definitions for COVID-19 according to the Ministry of Health and Social Protection of Colombia.

**Additional file 6.** Definition of the categories of the adherence variable.

**Additional file 7: Table.** Description of the variables associated with the state of health of the participants during the follow-up period.

**Additional file 8: Table.** Post hoc analysis of the primary outcome considering the adherence.

## Data Availability

Individual participant data will not be openly available. The data will be shared with investigators whose proposed use of the data has been approved by an ethical research committee (e.g., for individual participant data meta-analysis). In particular, data that underlie the results reported in this article, after de-identification (text, tables, figures, and appendices), will be shared. No other documents will be available. Proposals should be directed to an-rami2@uniandes.edu.co; to gain access, data requestors will need to sign a data access agreement. The data will be available immediately after publication for up to 5 years on a third-party website that will be shared only with proposals received and approved via e-mail.
