# Supplementary material for: Effectiveness and adherence to closed face shields in the prevention of COVID-19 transmission: a non-inferiority randomized controlled trial in a middle-income setting (COVPROSHIELD)
Source: Trials. 2022 Aug 20;23:698. doi: 10.1186/s13063-022-06606-0 (PMC9391623; doi:10.1186/s13063-022-06606-0)
Supplement: Supplementary file 2 — Additional file 2. Description of the face shield implemented during the intervention. [file 13063_2022_6606_MOESM2_ESM.pdf]

S2 File. Description of the face shield implemented during the intervention

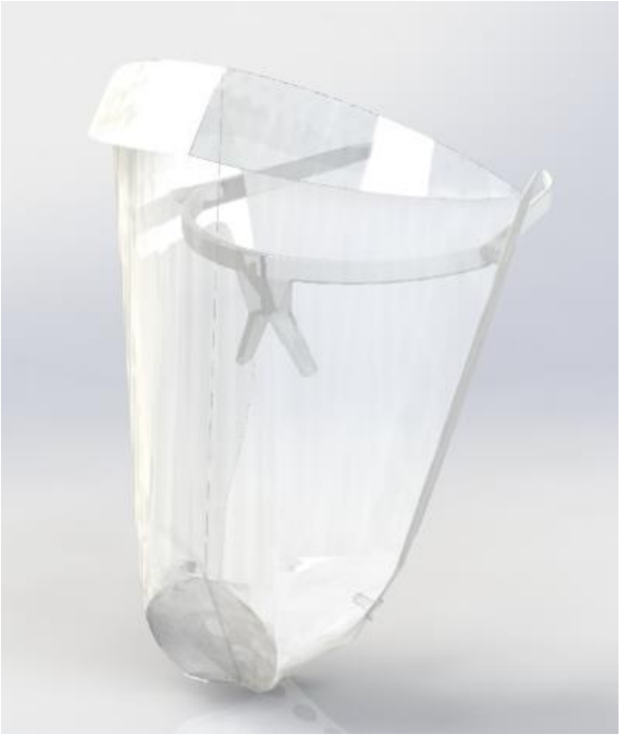

Figure 1. Ideplas ® Face shield Prototype.

The Ideas design is defined by its tight and narrow fit at the bottom and side, which protect from drafts approaching from below and at an angle. Its main shortcoming is its limited ventilation, which could cause the face shield to fog up with prolonged use. However, when combined with the face mask, poor ventilation and unpredictable drafts can be mitigated. The visor at the top of the protector does not make a seal with the forehead, leaving space for air to enter from the top. Contact with the forehead prevents sweat droplets (which may have absorbed virus particles) from traveling down the face to the eyes or mouth. Its design is suitable for protecting the user from direct air currents but not to protect others from the user.

Reference

1. Medina A, Ewing D, Stauff JR. Studies of the Effectiveness of Face Shields against Airborne Particulate Matter by Flow Simulation. Yale University.
