# Supplementary material for: Effectiveness and adherence to closed face shields in the prevention of COVID-19 transmission: a non-inferiority randomized controlled trial in a middle-income setting (COVPROSHIELD)
Source: Trials. 2022 Aug 20;23:698. doi: 10.1186/s13063-022-06606-0 (PMC9391623; doi:10.1186/s13063-022-06606-0)
Supplement: Supplementary file 3 — Additional file 3. Recorded educational intervention during the follow-up period. [file 13063_2022_6606_MOESM3_ESM.pdf]

S3 File. Recorded educational intervention during the follow-up period

To see in: https://youtu.be/tR3v2chVXpA
