# Supplementary material for: Effectiveness and adherence to closed face shields in the prevention of COVID-19 transmission: a non-inferiority randomized controlled trial in a middle-income setting (COVPROSHIELD)
Source: Trials. 2022 Aug 20;23:698. doi: 10.1186/s13063-022-06606-0 (PMC9391623; doi:10.1186/s13063-022-06606-0)
Supplement: Supplementary file 4 — Additional file 4. Questionnaire to evaluate the state of health and proper use of the intervention during the follow-up period. [file 13063_2022_6606_MOESM4_ESM.pdf]

S4 File. Questionnaire to evaluate the state of health and proper use of the intervention during the follow-up period

Evaluation of the state of health

1. Are you in isolation because you have had contact with someone positive for COVID-19?
2. Are you hospitalized or isolated for COVID-19?
3. In the last seven days, have you had any symptoms such as Dry cough, Fever, Sore throat, Difficulty breathing, Extreme fatigue/tiredness/adynamia, Diarrhea, Alterations in the sense of smell or alterations in the sense of taste, Confusion of recent onset? If yes:
    a. What symptoms? (date of onset of symptoms)
    b. Have you been: Residing, working, or traveling in a high-risk area for transmission of the virus, such as a closed residential facility or a humanitarian facility, such as a displaced person camp, prison, or similar facility, at any time in the past four days? Or In close contact with someone infected with COVID-19, i.e., shared in the space of fewer than two meters and for more than 15 minutes without personal protective equipment or with equipment for more than two hours.
    c. Because of your symptoms, have you consulted an emergency line, outpatient or priority consultation, private physician, occupational physician, or have you taken any medication (if yes, which one)?
    d. Have you been tested for COVID-19 due to your symptoms?
        i. If yes: who performed the test and which type of test was it (RT-PCR, Antigen, Serological). When? Which was the result?
    e. Have the people you live with been tested for COVID-19 in the last seven days?
        i. If yes: who performed the test and which type of test was it (RT-PCR, Antigen, Serological). When? Which was the result?

Evaluation of the adherence to the intervention

Thinking About the last week:

a. What days did you go out to work, visit friends, or go shopping?
    a. Those days that you did go out did you wear the personal protective equipment (PPE) assigned. If not, why?
b. On those days, you ever took off the PPE, if so why, and how many times.
c. Thinking about the last day of use, on average, how many hours per day did you use the devices?
d. Do you use the PPE indoors?

Attitudes towards the use

Considering the categories of totally disagree, disagree, neither agree nor disagree, agree or totally agree, do you consider that:

a. The use of the PPE assigned increases splash protection:
b. The use of the PPE assigned prevents others from contracting the virus:
c. People close to you consider it strange to wear the PPE assigned:
d. It is rare for someone to wear the PPE assigned in public:

E. Give an example of a time when you felt more protected by wearing the PPE assigned during this week:

Barriers related to the use

a. Did wearing the PPE cause you discomfort? Yes/No. If yes, what was the reason?
b. How do you rate the PPE assigned comfort (very comfortable, uncomfortable, neither comfortable nor uncomfortable, comfortable, very comfortable)?
c. On a scale of 1 to 10, 10 being the most uncomfortable possible, how uncomfortable was it for you to wear the PPE assigned?
d. Considering the categories not at all, a little, quite a bit, or a lot, how much did you feel wearing the PPE assigned the last week:
    i. Hot/humidity:
    ii. Difficulty breathing
    iii. Difficulty visualizing
    iv. Difficulty in communicating
    vi. Sensation of claustrophobia
    vii. Feeling of embarrassment or being judged for wearing it
    viii. Sense of suffocation

Considering the categories of totally disagree, disagree, neither agree nor disagree, agree, or totally agree

i. Do you consider that the PPE assigned are easy to use, take off, and adjust?
ii. Do you consider that the use of the PPE limits communication with others by limiting facial expression, visibility, or vocalization?

In the intervention group: During the last week, when you used the face shield, did you clean it with soap and water or alcohol at the end of the day? (all times, sometimes, once, never)

In both groups: Did you change the face mask daily? If not, why?

Continuity of use

a. Do you plan to continue using the PPE assigned after the study is completed: if not, why?

b. From 1 to 10, with 1 being very unlikely and 10 being very likely. How likely are you to recommend the use of the PPE assigned to your friends or family members?
c. Do you have any additional comments regarding the advantages or disadvantages of using the PPE assigned?
