# Supplementary material for: Effectiveness and adherence to closed face shields in the prevention of COVID-19 transmission: a non-inferiority randomized controlled trial in a middle-income setting (COVPROSHIELD)
Source: Trials. 2022 Aug 20;23:698. doi: 10.1186/s13063-022-06606-0 (PMC9391623; doi:10.1186/s13063-022-06606-0)
Supplement: Supplementary file 5 — Additional file 5. Case definitions for COVID-19 according to the Ministry of Health and Social Protection of Colombia. [file 13063_2022_6606_MOESM5_ESM.pdf]

S5 File. Case definitions for COVID-19 according to the Ministry of Health and Social Protection of Colombia

1. Asymptomatic case (Definition 5 INS pg. 10): Close contact of a confirmed COVID-19 case that has not manifested symptoms in the first seven days after the last unprotected exposure.

2. Suspected case: ARI (Definition 3.2 INS) person with a quantified fever greater than 38 ° C and cough of no more than seven days of evolution, that requires in-hospital management in the emergency department or observation, with an emphasis on:
- Patients over 60 years of age and with comorbidities (diabetes, cardiovascular disease (includes hypertension and stroke), failure kidney, HIV or other immunodeficiencies, cancer, autoimmune diseases, hypothyroidism, use of corticosteroids or immunosuppressants, COPD and asthma, malnutrition (obesity and malnutrition) and smokers.
- Health workers, including administrative, police, and military forces, employees of social protection services of children, adolescents or the elderly, employees of prisons, police stations, and URI, ethnic groups (indigenous, black communities, Afro-Colombians, Raizales, Palenqueros, and Rom), people in a street situation, people deprived of liberty, people in high mobility by their work occupation, internal Colombian migrants.

3. Suspicious case: SARI (Definition 3.1 INS) person with a quantified fever greater than 38 ° C and cough of no more than ten days of evolution, that requires in-hospital management in the emergency department, observation, hospitalization, or ICU, with an emphasis on
- Patients over 60 years of age and with comorbidities (diabetes, cardiovascular disease (includes hypertension and stroke), failure kidney, HIV or other immunodeficiencies, cancer, autoimmune diseases, hypothyroidism, use of corticosteroids or immunosuppressants, COPD and asthma, malnutrition (obesity and malnutrition) and smokers.
- Health workers, including administrative, police, and military forces, employees of social protection services of children, adolescents or the elderly, employees of prisons, police stations, and URI, ethnic groups (indigenous, black communities, Afro-Colombians, Raizales, Palenqueros, and Rom), people in a street situation, people deprived of liberty, people in high mobility by their work occupation, internal Colombian migrants.

4. Probable case: Unusual SARI 348 (Definition 1 INS) Acute respiratory infection (SARI, due to quantified fever greater than or equal to 38 ° C and cough, with unusual or unexpected clinical course, especially sudden deterioration despite proper treatment, which does require hospitalization) AND
- History of international or national travel to areas with local community transmission in the four days before the symptoms start.
- Practicing health/hospital worker who has had close contact with a confirmed or probable case of COVID-19
- History of close contact in the last 14 days with a confirmed case of SARI associated with COVID-19.

5. Probable case: COVID-19 (Definition 2 INS) Quantified fever greater than 38 ° C, cough, respiratory distress, odynophagia and or fatigue, asthenia, AND at least one of the following
- History of international or national travel to areas with local community transmission in the four days before the start of the symptoms.
- Practicing health/hospital worker who has had close contact with a confirmed or probable case of COVID-19.
- People in high mobility due to their work occupation who have had close contact with a confirmed or probable case

by COVID-19.
- History of close contact in the last four days with a confirmed case of SARI associated with COVID-19.

6. Case of probable death due to COVID (Definition 4 INS): All deaths due to severe acute respiratory infection with a clinical picture of unknown etiology during the consultation, admission, observation, hospitalization, or at home (SARI 348), with emphasis on
- Patients over 60 years of age and with comorbidities (diabetes, cardiovascular disease (includes hypertension and stroke), failure kidney, HIV or other immunodeficiencies, cancer, autoimmune diseases, hypothyroidism, use of corticosteroids or immunosuppressants, COPD and asthma, malnutrition (obesity and malnutrition) and smokers.
- Health workers, including administrative, police, and military forces, employees of social protection services of children, adolescents, or the elderly, employees of prisons, police stations and URI, ethnic groups (indigenous, black communities, Afro-Colombians, Raizales, Palenqueros, and Rom), people in a street situation, people deprived of liberty, and high mobility by their work occupation, internal Colombian migrants.

7. Confirmed case (INS 5 pg. 16). Asymptomatic laboratory-confirmed case: the probable case that meets any of the following criteria:
- RT-PCR for SARS-CoV-2 in patients with less than 14 days from the date of the last exposure not protected with confirmed case for COVID-19.

8. Confirmed case 3.2 Mild Acute Respiratory Infection (equivalent to Influenza-Like Disease) (Code 345) confirmed by the laboratory: the suspected case that meets any of the following criteria:
- RT-PCR positive for SARS-CoV-2 in patients less than 14 days from the date of symptom onset. In the cases where the RT-PCR is negative, the confirmation of a case that manifests symptoms 11 days ago or more with:
o Positive serological test for IgM / IgG antibodies with 11 days or more from the date of the onset of symptoms (The Serological tests used must comply with the characteristics of sensitivity and specificity, concordance with PCR, and sample size required by the Guidelines for the use of tests diagnoses of SAR-COV-2 (COVID-19) in Colombia from the Ministry of Health and Social Protection). With the available evidence, the use of serological tests in an asymptomatic population is not recommended except that the inquiry establishes previous symptomatic periods.

9. Confirmed case 2. Mild or moderate acute respiratory infection by a new virus, with confirmed home management (Code 346) by laboratory: probable case that meets any of the following criteria:
- RT-PCR positive for SARS-CoV-2 in patients less than 14 days from the date of symptom onset. In the In cases where the RT-PCR is negative, the confirmation of a case that manifests symptoms 11 days ago or more with:
o Positive serological test for IgM / IgG antibodies with 11 days or more from the date of the onset of symptoms (The serological tests used must comply with the characteristics of sensitivity and specificity, concordance with PCR, and sample size required by the Guidelines for the use of tests diagnoses of SAR-COV-2 (COVID-19) in Colombia from the Ministry of Health and Social Protection). With the available evidence, the use of serological tests in an asymptomatic population is not recommended except that the inquiry establishes previous symptomatic periods.

10. Confirmed case 3.1 Serious Acute Respiratory Infection (Code 345) confirmed by the laboratory: the suspected case that complies with any of the following criteria:
- RT-PCR positive for SARS-CoV-2 in patients less than 14 days from the date of symptom onset. In the In cases where the RT-PCR is negative, the case can be evaluated with the following elements:
o Positive serological test for IgM / IgG antibodies with 11 days or more from the date of the onset of symptoms (The

Serological tests used must comply with the characteristics of sensitivity and specificity, concordance with PCR, and sample size required by the Guidelines for the use of tests diagnoses of SAR-COV-2 (COVID-19) in Colombia from the Ministry of Health and Social Protection). With the available evidence, the use of serological tests in asymptomatic populations is not recommended except that the inquiry establishes previous symptomatic periods.
o Clinical criteria such as pulmonary radiological findings (parenchymal glass opacities
frosting or peripheral consolidation and predominantly baseline), increased D-dimer, CRP, ferritin, or LDH, as well
such as lymphopenia and thrombocytopenia.
o Characteristics such as a population at risk or vulnerable.

11. Confirmed case 1 SARI-Unusual 1: Laboratory confirmed case: the probable case that meets one of the following
criteria:
- RT-PCR positive for SARS-CoV-2 in patients less than 14 days from the date of onset of symptoms. In the In cases where the RT-PCR is negative, the case can be evaluated with the following elements:
o Positive serological test for IgM / IgG antibodies with 11 days or more from the date of the onset of symptoms (The serological tests used must comply with the characteristics of sensitivity and specificity, concordance with PCR, and sample size required by the Guidelines for the use of tests SAR-COV-2 (COVID-19) diagnoses in Colombia from the Ministry of Health and Social Protection)
o Clinical criteria such as pulmonary radiological findings (parenchymal glass opacities frosting or peripheral consolidation and predominantly baseline), increased D-dimer, CRP, ferritin, or LDH, as well such as lymphopenia and thrombocytopenia.

12. Confirmed case 4. Death by COVID-19 (Code 348) confirmed by the laboratory: probable case that meets one of the following criteria:
- RT-PCR positive for SARS-CoV-2 in patients with less than 14 days from the date of onset of symptoms, in test
pre-mortem or post-mortem (up to 6 hours) in respiratory or tissue samples. In cases where RT-PCR is negative
(False-negative results can occur due to: the anatomical site where the sample is collected respiratory (nasopharyngeal, oropharynx, tracheal or bronchial), the time in relation to the date of onset of symptoms (very close today zero or day fourteen), the viral transport medium, refrigeration and the amount of respiratory secretion, among others (11. Laboratory Guidelines for the Detection and Diagnosis of Infection with the COVID-19 Virus, Pan American Health Organization), the case can be assessed with the following elements:
- Positive serological test for IgM / IgG antibodies with 11 days or more from the date of the onset of symptoms (Tests
The serological tests used must comply with the characteristics of sensitivity and specificity, concordance with PCR and sample size required by the Guidelines for the use of SAR-COV-2 (COVID-19) diagnostic tests in Colombia from the Ministry of Health and Social Protection). With the available evidence, serological tests in asymptomatic population are used unless the investigation establishes symptomatic periods previous.
- Clinical criteria such as pulmonary radiological findings (ground glass parenchymal opacities or peripheral consolidation and predominantly baseline), increased D-dimer, CRP, ferritin, or LDH, as well as lymphopenia and
thrombocytopenia.
- The characteristics as a population at risk or vulnerable.

13. Confirmed case 4. Death from COVID-19 (Code 348) Confirmed case due to epidemiological link: probable case that complies with one of the following criteria:
- The deceased person had previous close contact with a confirmed case of COVID-19 between 1 and 14 days before the start of the symptom.

- At least one close contact of the deceased person is detected as a confirmed case of COVID-19 between 1 and 14 days after the last unprotected contact.

14. Discarded case: Person who meets the definition of the probable case (in this annex) and has a negative result for
novel coronavirus 2019 (COVID-19) using real-time RT-PCR.

Taken and adapted from: https://www.minsalud.gov.co/sites/rid/Lists/BibliotecaDigital/RIDE/VS/ED/VSP/pspm01-man-lineamientos-formulacion-casos-vsp.pdf
