# Supplementary material for: Effectiveness and adherence to closed face shields in the prevention of COVID-19 transmission: a non-inferiority randomized controlled trial in a middle-income setting (COVPROSHIELD)
Source: Trials. 2022 Aug 20;23:698. doi: 10.1186/s13063-022-06606-0 (PMC9391623; doi:10.1186/s13063-022-06606-0)
Supplement: Supplementary file 6 — Additional file 6. Definition of the categories of the adherence variable. [file 13063_2022_6606_MOESM6_ESM.pdf]

S6 File. Definition of the categories of the adherence variable.

Category	Follow-up call	Follow-up call	Follow-up call
High adherence			
Medium-high adherence			
Medium-high adherence			
Medium-high adherence			
Medium adherence			
Medium-low adherence			
Medium-low adherence			
Medium-low adherence			
Low adherence			
Low adherence			

Total adherence: the proper daily use of the surgical mask or the closed face-shield with surgical mask (every time the participant left his/her home, removed only for eating, driving alone/riding a bicycle or while being alone). In the case of the closed face shield, it had to be washed or disinfected with alcohol daily, and the face mask must be changed every day.

Partial adherence: using the surgical mask or the closed face-shield with surgical mask whenever participants went out but reported removal of surgical mask or the closed face-shield with surgical mask for a reason different from those recommended cleaning/changing of the device properly. Also, partial adherence included using a surgical mask or the closed face-shield with surgical mask whenever they went out without removing the surgical mask or the closed face-shield with surgical mask, but without cleaning/changing the devices appropriately.

Non-proper adherence: using the surgical mask or the closed face-shield with surgical mask whenever the participant went out from their homes but with the removal of surgical mask or the face-shield with surgical mask for a reason different from those recommended and not proper cleaning/changing of the assigned device. Non-adherence was also defined if the participants reported that they went outsides their homes without using the surgical mask or the closed face-shield + surgical mask.
