# Supplementary material for: Effectiveness and adherence to closed face shields in the prevention of COVID-19 transmission: a non-inferiority randomized controlled trial in a middle-income setting (COVPROSHIELD)
Source: Trials. 2022 Aug 20;23:698. doi: 10.1186/s13063-022-06606-0 (PMC9391623; doi:10.1186/s13063-022-06606-0)
Supplement: Supplementary file 7 — Additional file 7: Table. Description of the variables associated with the state of health of the participants during the follow-up period. [file 13063_2022_6606_MOESM7_ESM.pdf]

S7 Table 6. Participants who reported at least one symptom related to COVID-19 during the follow-up period (Dry cough, Fever, Sore throat, Shortness of breath, Fatigue/extreme tiredness, Diarrhea, Alterations of smell or taste)

Table 6. Participants who reported at least one symptom related to COVID-19 during the follow-up period (Dry cough, Fever, Sore throat, Shortness of breath, Fatigue/extreme tiredness, Diarrhea, Alterations of smell or taste)

	Week 1<br>N (%)	Week 2<br>N (%)	Week 3<br>N (%)
Active control group (n=142)			
**Reported any symptom**	**4(2·8%)**	**3(2·1%)**	**4(2·9%)**
Cough	3(75%)	1(33·3%)	1(25%)
Fever	0(0%)	0(0%)	0(0%)
Sore throat	1(25%)	3(100%)	2(50%)
Shortness of breath	1(25%)	0(0%)	1(25%)
Fatigue/extreme tiredness	0(0%)	2(66·7%)	0(0%)
Diarrhea	0(0%)	0(0%)	0(0%)
Alterations of taste or smell	0(0%)	1(33·3%)	0(0%)
Epidemiological nexus	1(25%)	1(33·3%)	2(50%)
Intervention group (n=141)			
**Reported any symptom**	**3(2·1%)**	**2(1·5%)**	**2(1·4%)**
Cough	1(33·3%)	1(50%)	0(0%)
Fever	1(33·3%)	0(0%)	0(0%)
Sore throat	1(33·3%)	1(50%)	1(50%)
Shortness of breath	1(33·3%)	0(0%)	0(0%)
Fatigue/extreme tiredness	1(33·3%)	1(50%)	0(0%)
Diarrhea	1(33·3%)	1(50%)	0(0%)
Alterations of taste or smell	1(33·3%)	0(0%)	0(0%)
Epidemiological nexus	1(33·3%)	0(0%)	0(0%)
