# Supplementary material for: Effectiveness and adherence to closed face shields in the prevention of COVID-19 transmission: a non-inferiority randomized controlled trial in a middle-income setting (COVPROSHIELD)
Source: Trials. 2022 Aug 20;23:698. doi: 10.1186/s13063-022-06606-0 (PMC9391623; doi:10.1186/s13063-022-06606-0)
Supplement: Supplementary file 8 — Additional file 8: Table. Post hoc analysis of the primary outcome considering the adherence. [file 13063_2022_6606_MOESM8_ESM.pdf]

S8 Table. Post hoc analysis of the primary outcome considering the adherence

Table 7. Post hoc analysis for comparison of the primary outcome between groups

	Face shield + surgical face mask group N (%)	Surgical face mask group N (%)	Absolute risk difference	p-value*
**High adherence**	**(N=35)**	**(N=126)**	**(%) (CI 95%)**	
Primary composite outcome	1 (2·8)	3 (2·1)	1·28 (-4·64 – 7·21)	0·52
Positive RT-PCR test	1 (2·8)	1 (0·7)	2·06 (-3·66 – 7·79)	0·38
Positive Antibody test				
IgG	0(0)	2 (1·4)	-0·79 (-2·34 – 0·75)	0·78
IgM	0(0)	0(0)	-	-
**High and medium-high adherence**	**(N=103)**	**(N=141)**	**(%) (CI 95%)**	
Primary composite outcome	1(0·9)	3 (2·1)	-1·16 (-4·1 – 1·88)	0·43
Positive RT-PCR test	1 (0·9)	1 (0·7)	0·26 (-2·08 – 2·60)	0·66
Positive Antibody test				
IgG	0(0)	2 (1·4)	-1·42 (-3·37 – 0·53)	0·33
IgM	0(0)	0(0)	-	-
**High, medium-high, and medium adherence**	**(N=110)**	**(N=141)**	**(%) (CI 95%)**	
Primary composite outcome	1(0·9)	3 (2·1)	-1·21 (-4·18 – 1·75)	0·40
Positive RT-PCR test	1 (0·9)	1 (0·7)	0·19 (-2·05 – 2·45)	0·68
Positive Antibody test				
IgG	0(0)	2 (1·4)	-1·41 (-3·3 – 0·53)	0·31
IgM	0(0)	0(0)	-	-
